# Supplementary material for: Exploring the effects of hypoxia and reoxygenation time on hepatocyte apoptosis and inflammation
Source: PLoS One. 2024 Nov 21;19(11):e0310535. doi: 10.1371/journal.pone.0310535 (PMC11581257; doi:10.1371/journal.pone.0310535)

Fig.2

GAPDH


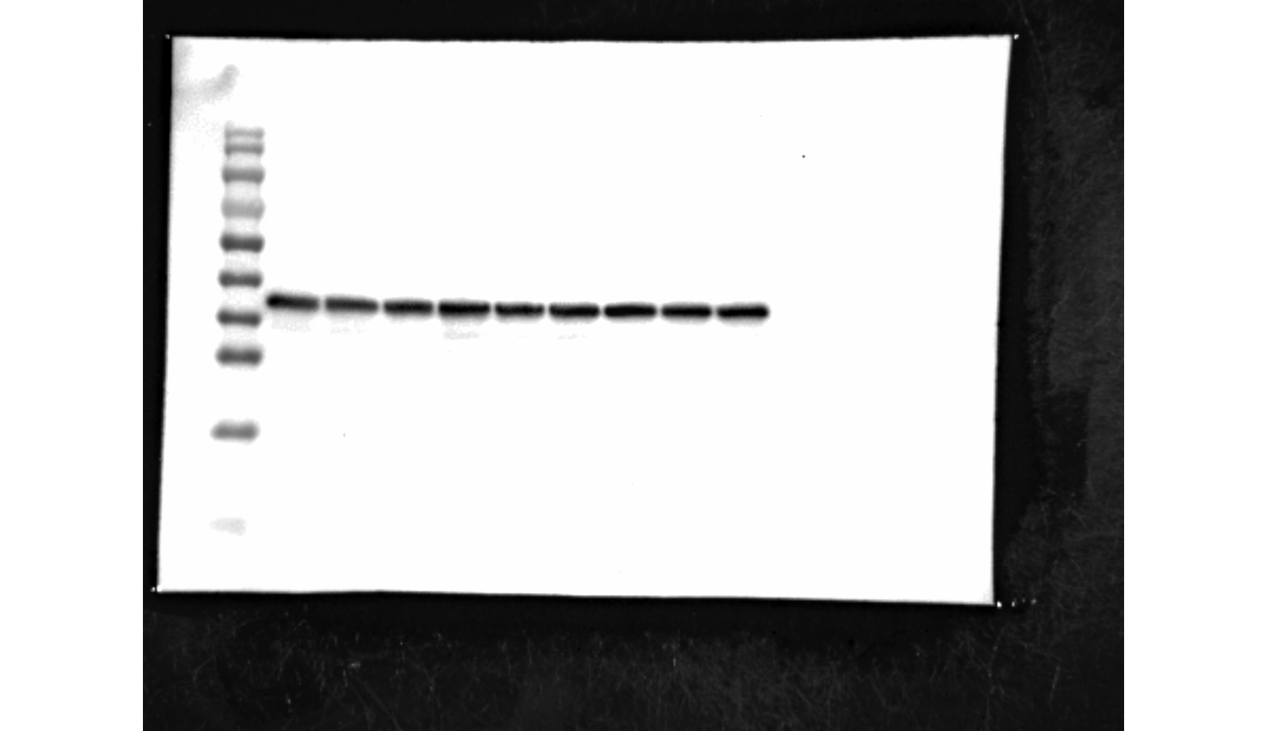


IL-6


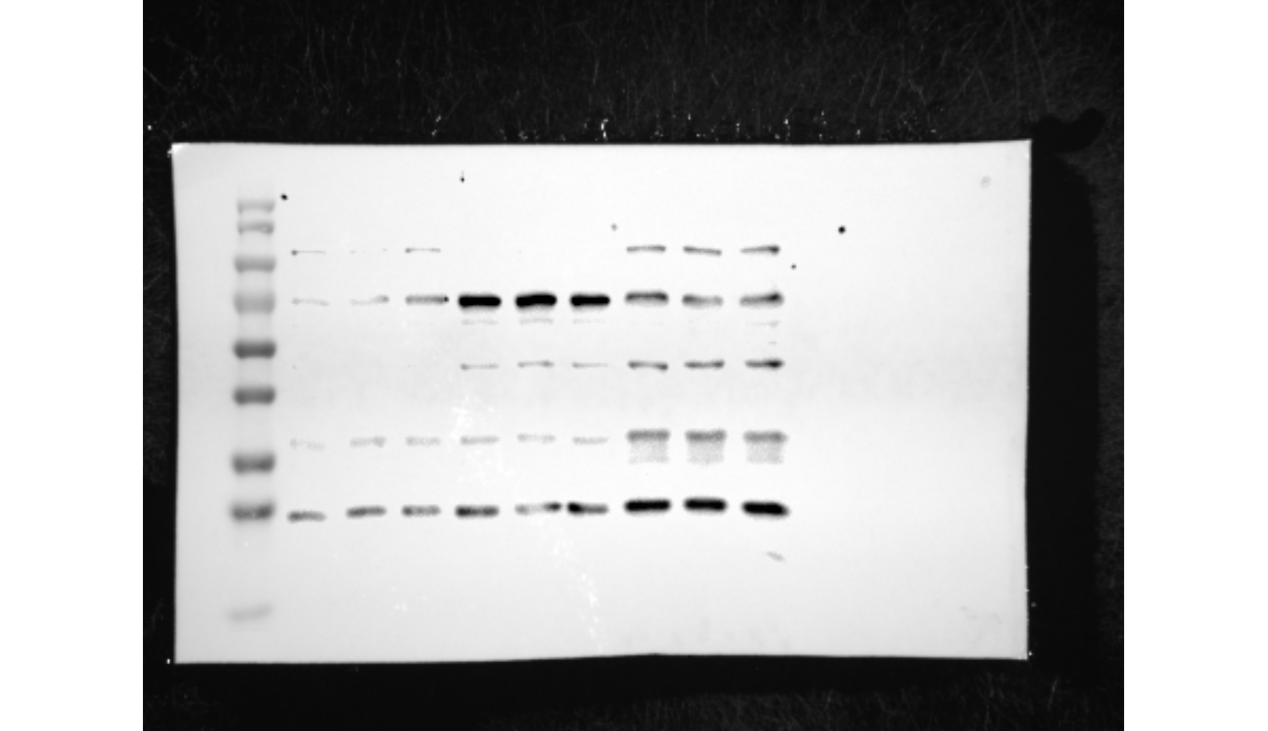


IL-1β


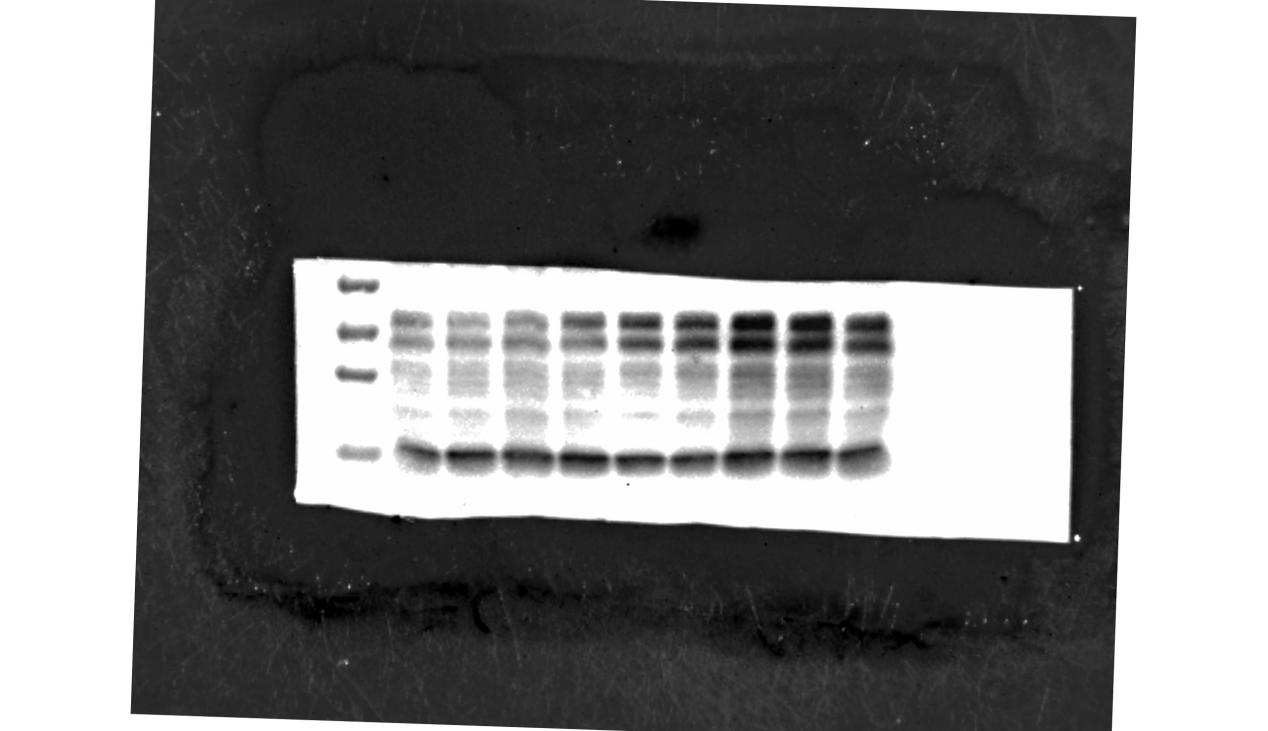


TNF-α


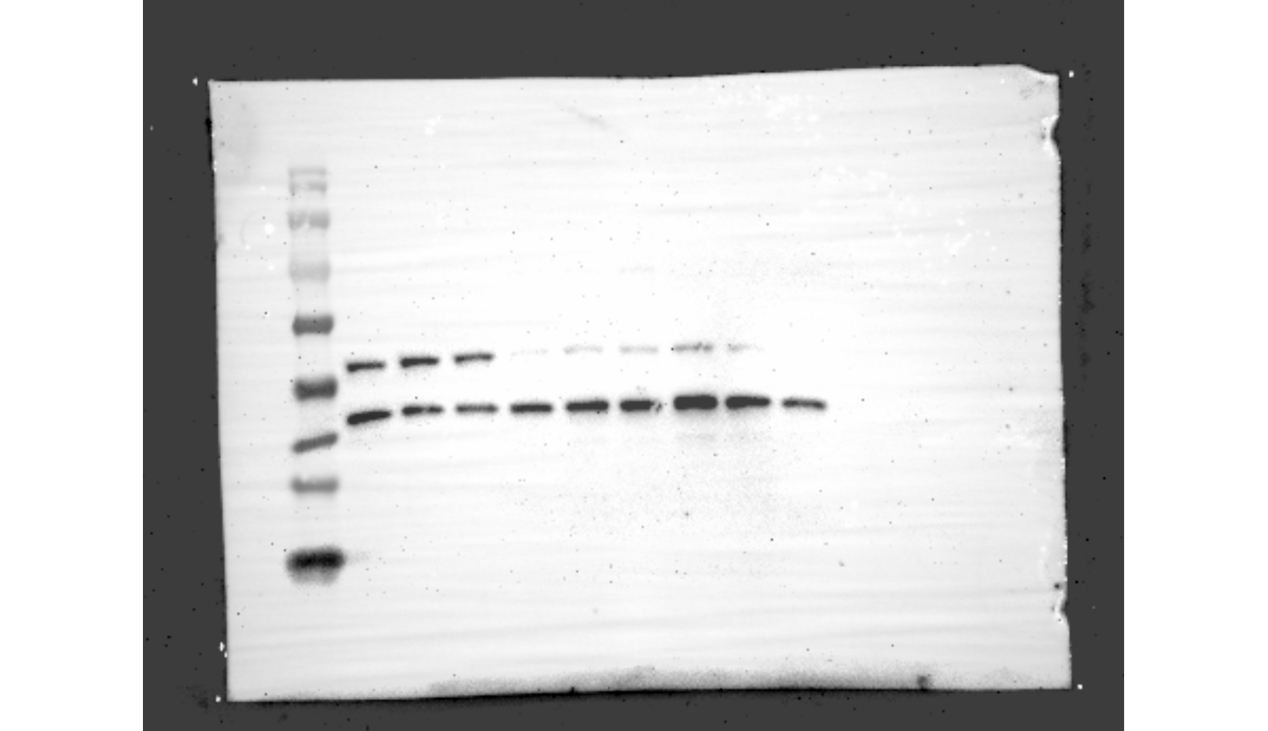


Fig.3

β-actin


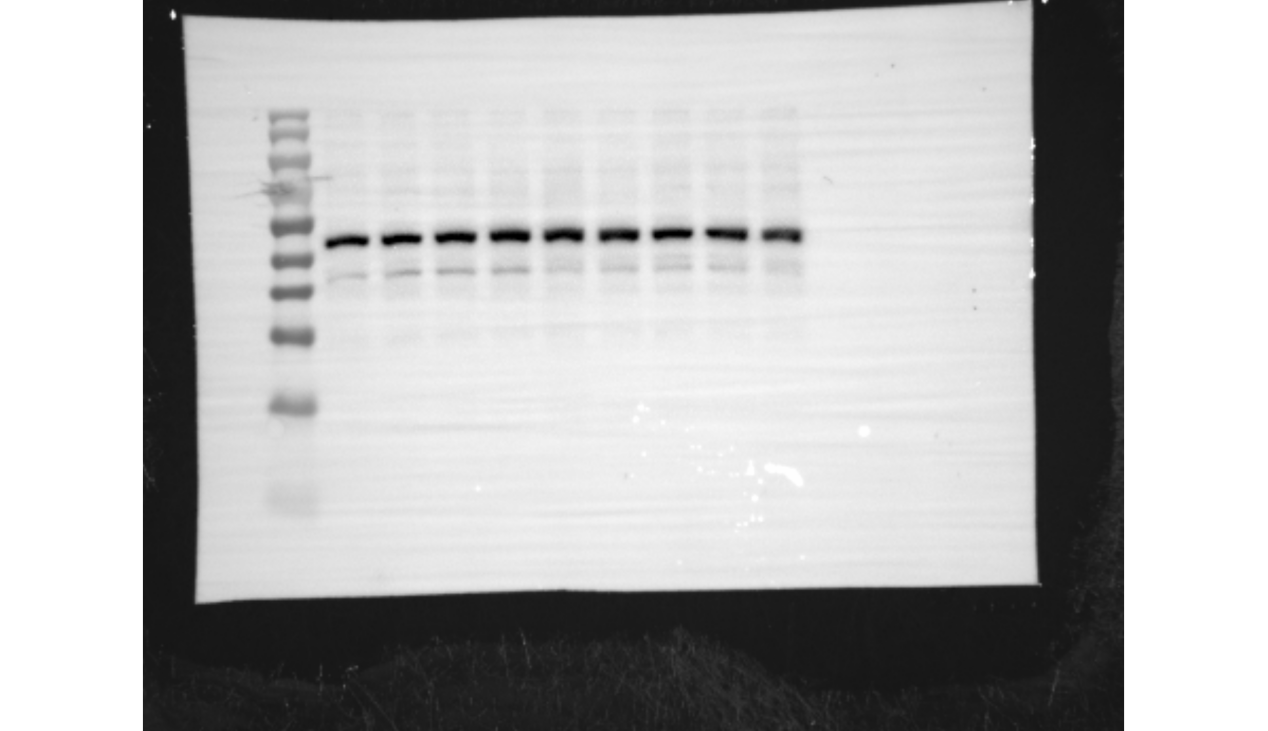


Bcl2


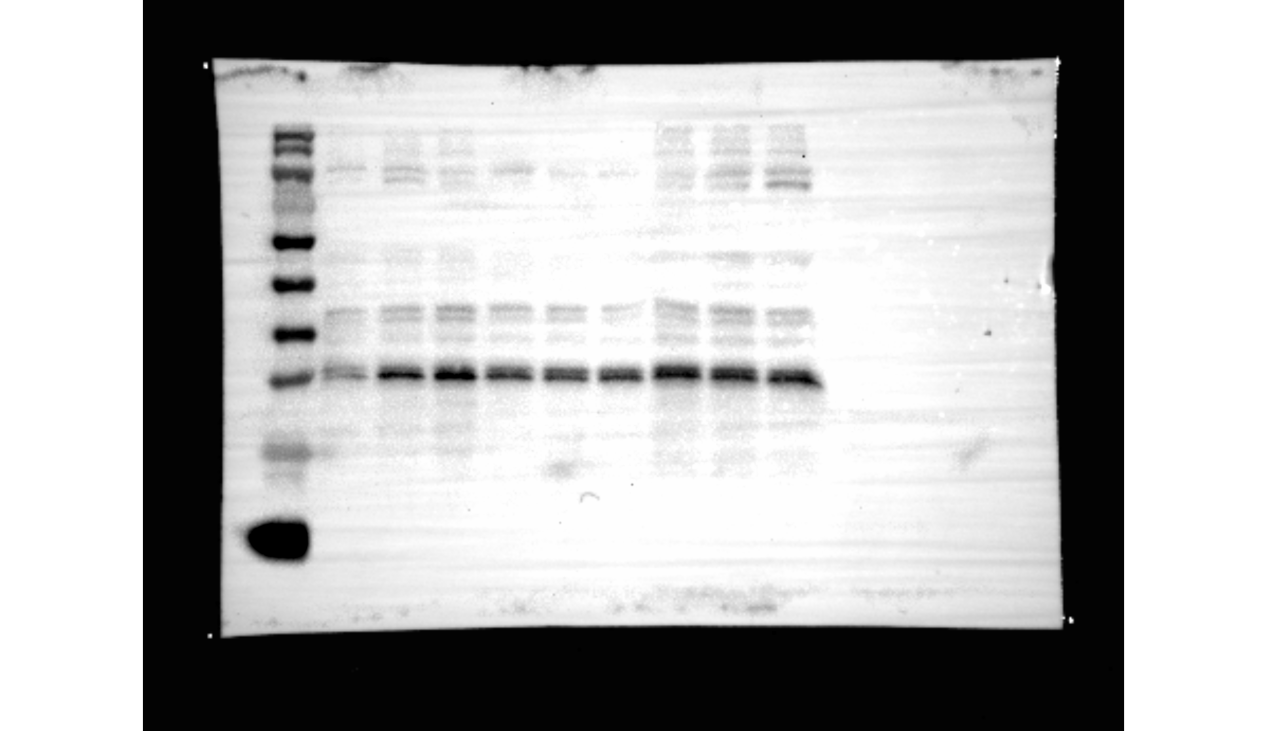


Caspase1


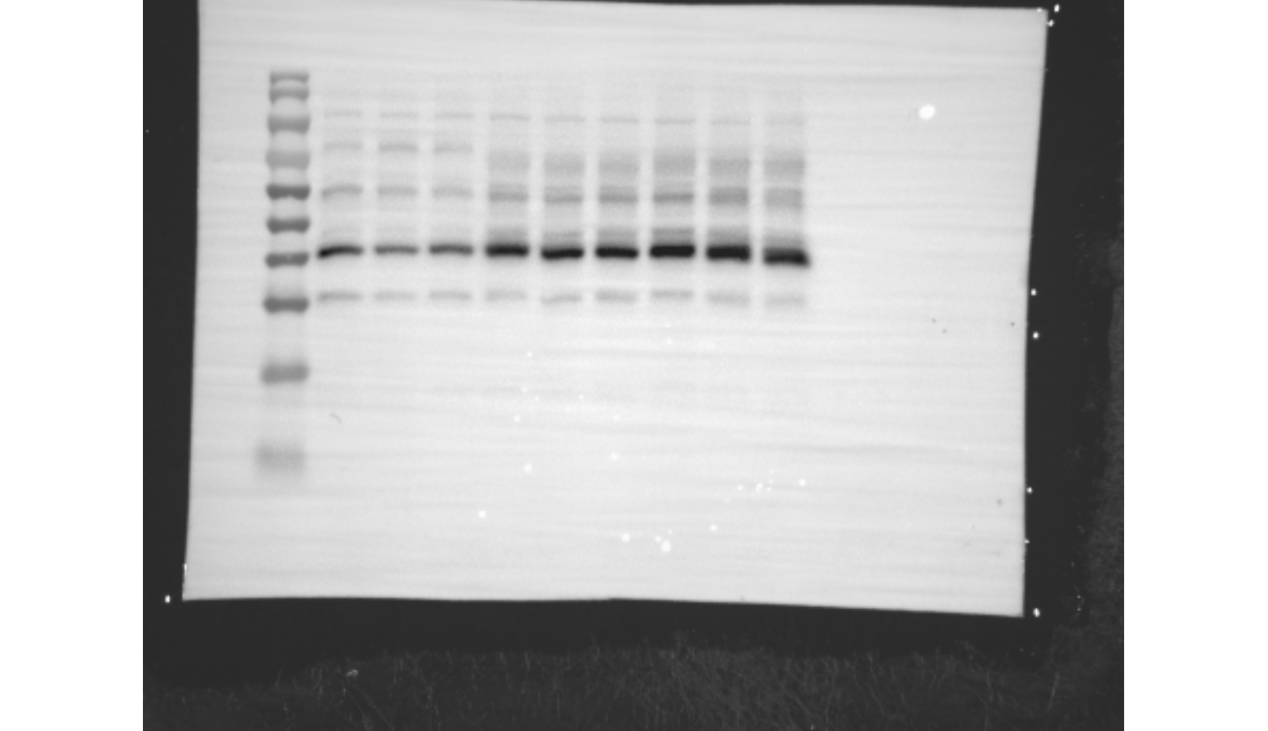


Caspase3


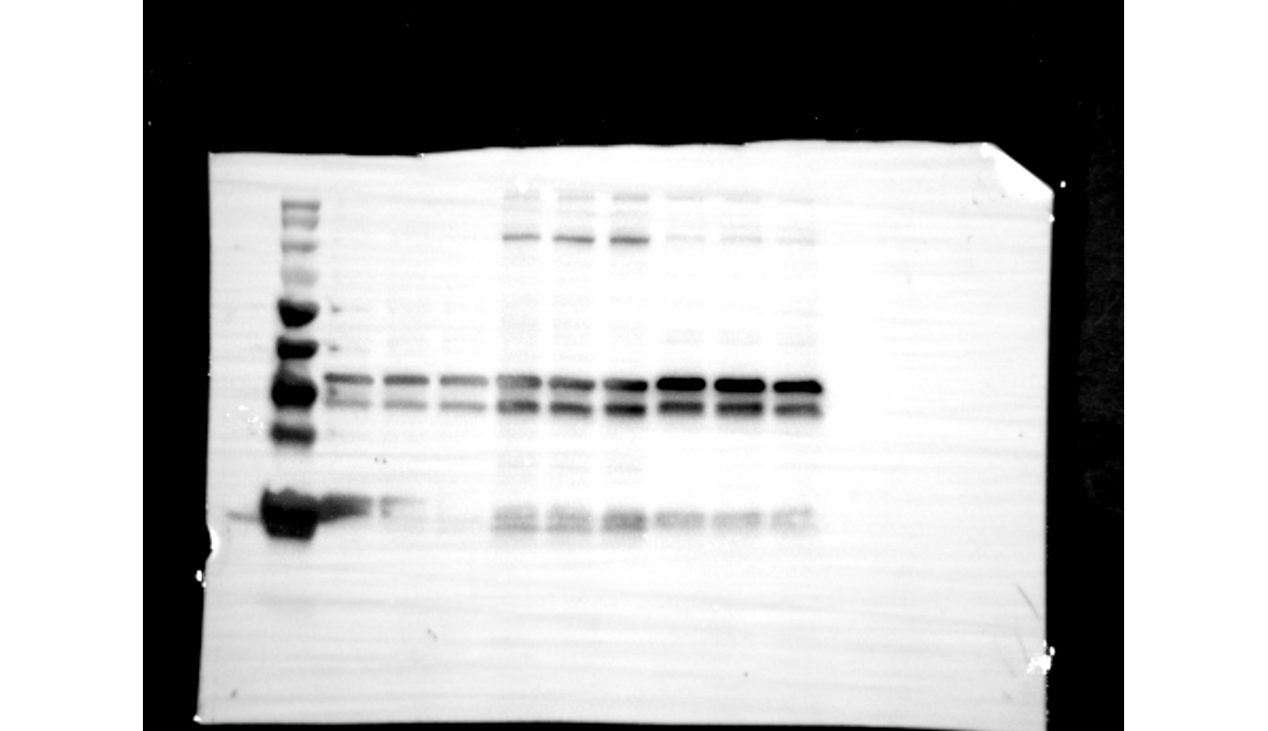


Fig.4

GAPDH


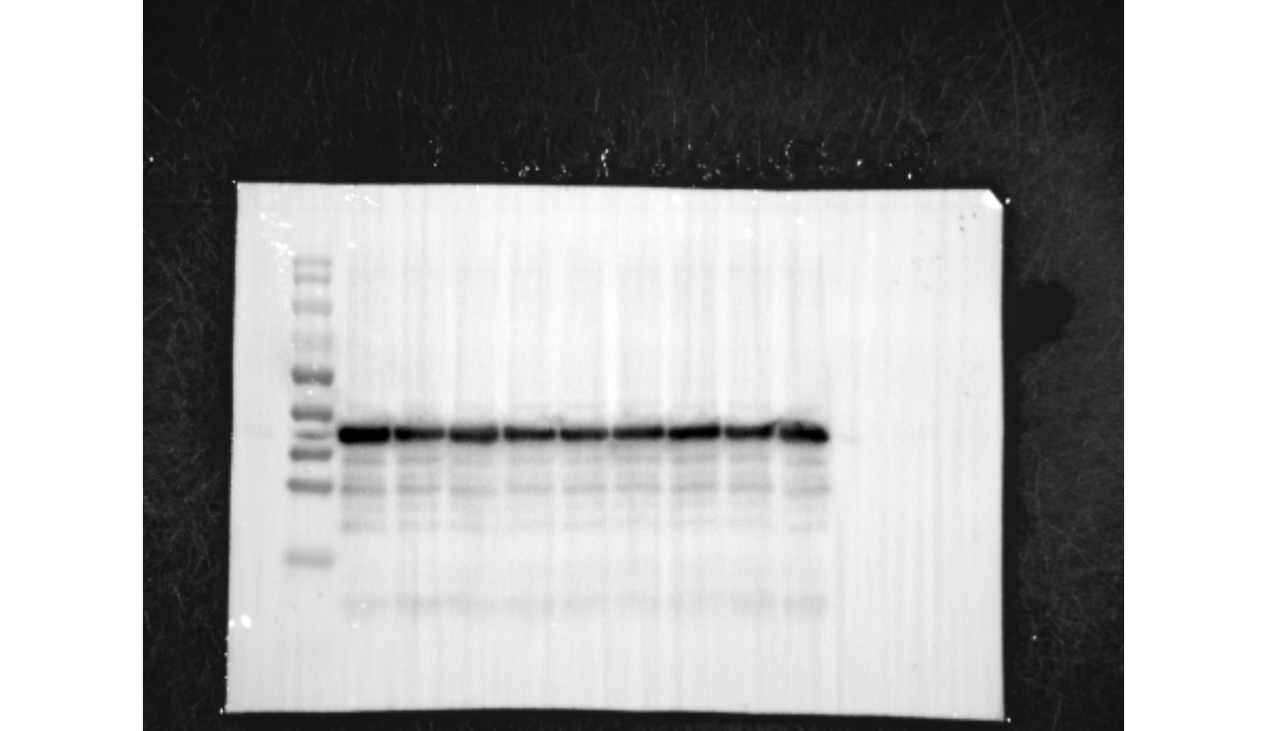


IL-6


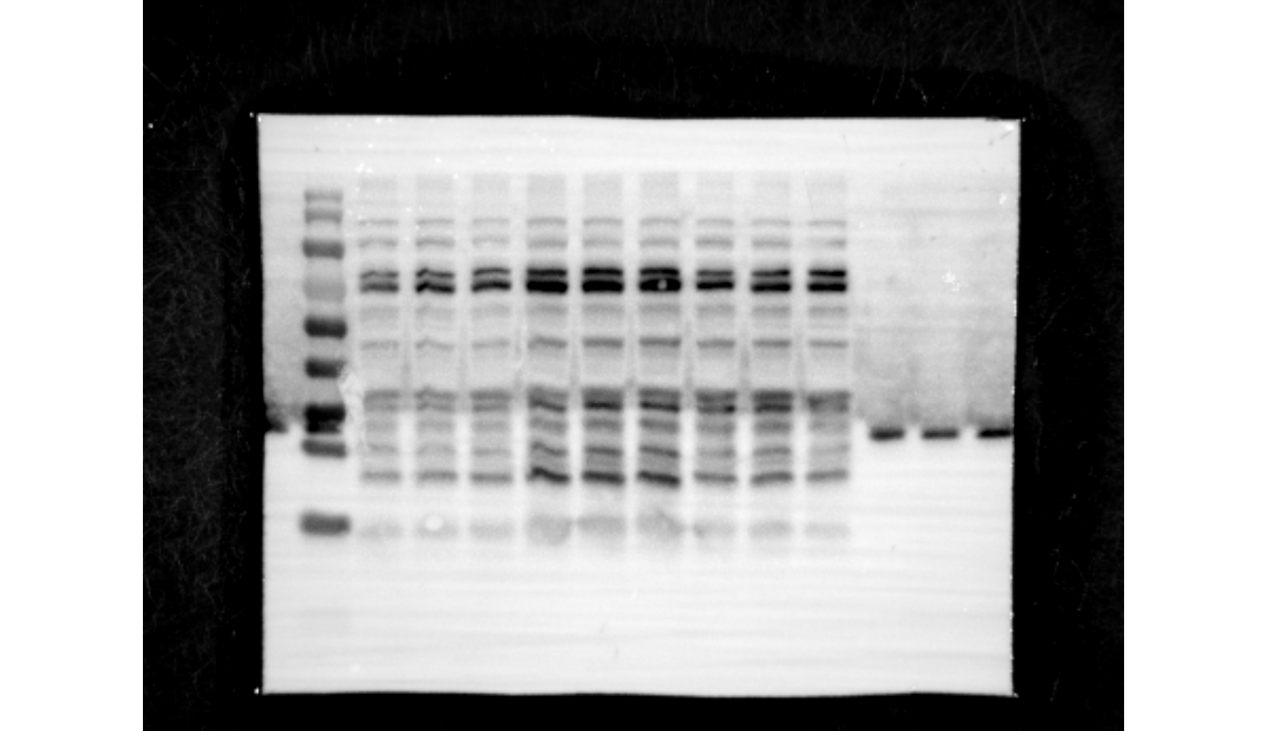


IL-1β


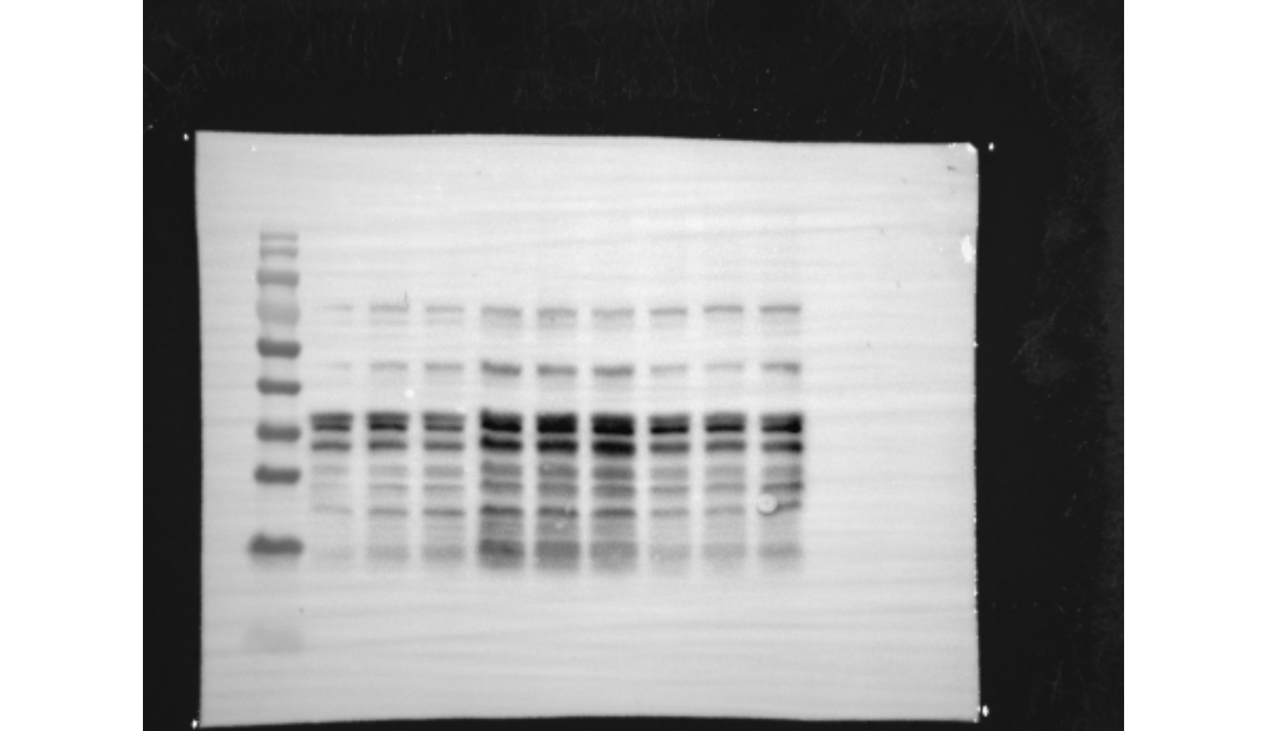


TNF-α


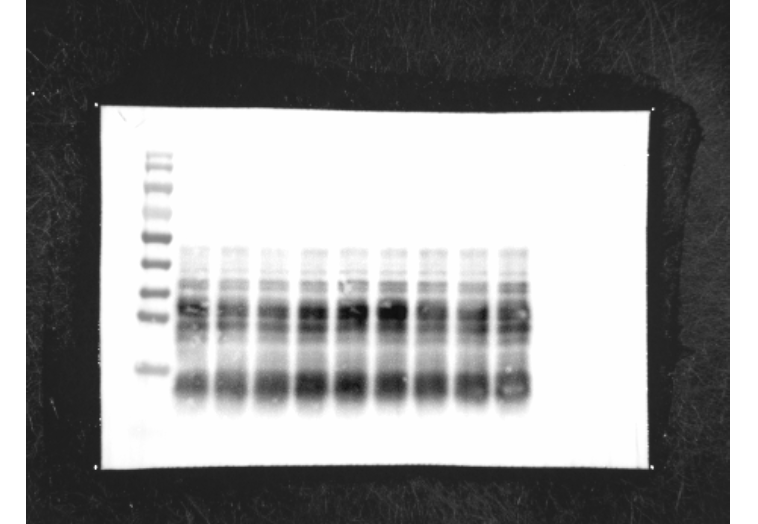


Fig.5

β-actin


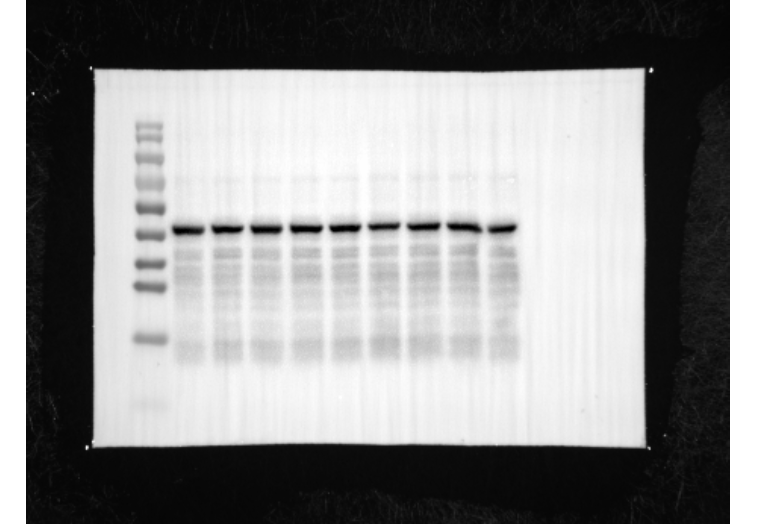


Bcl2


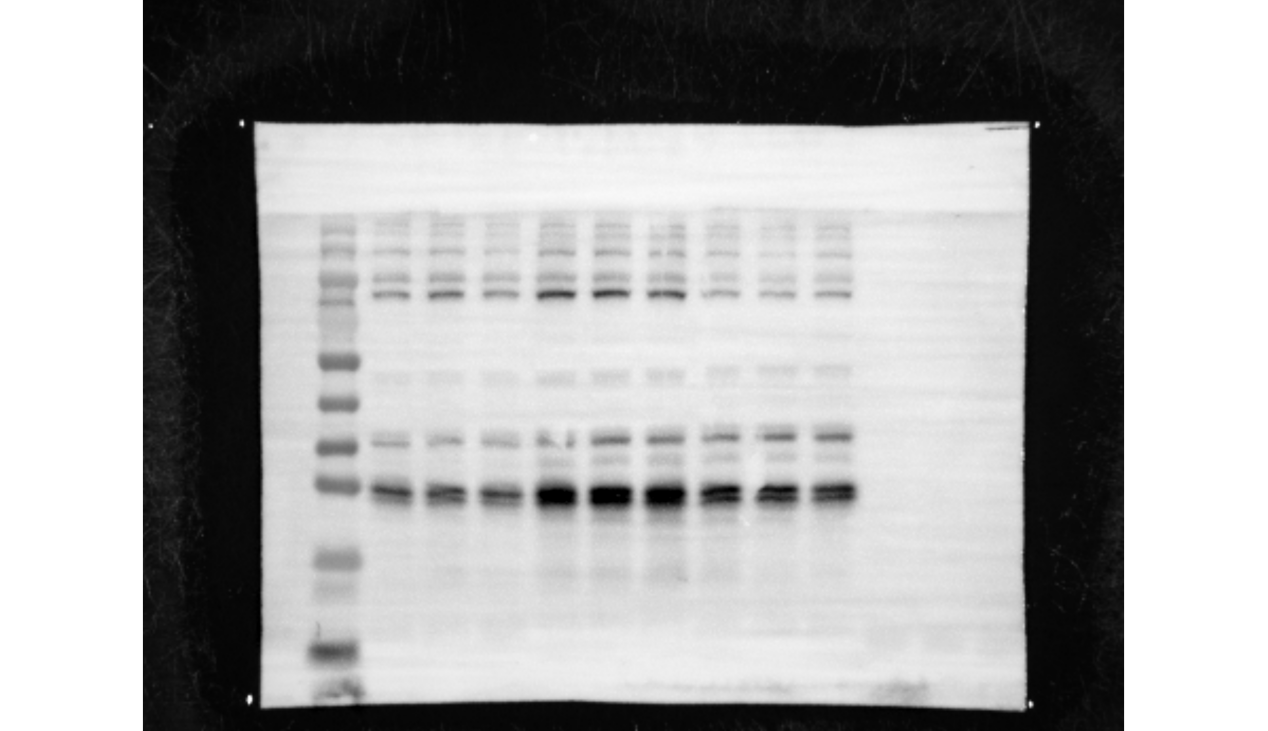


Caspase1


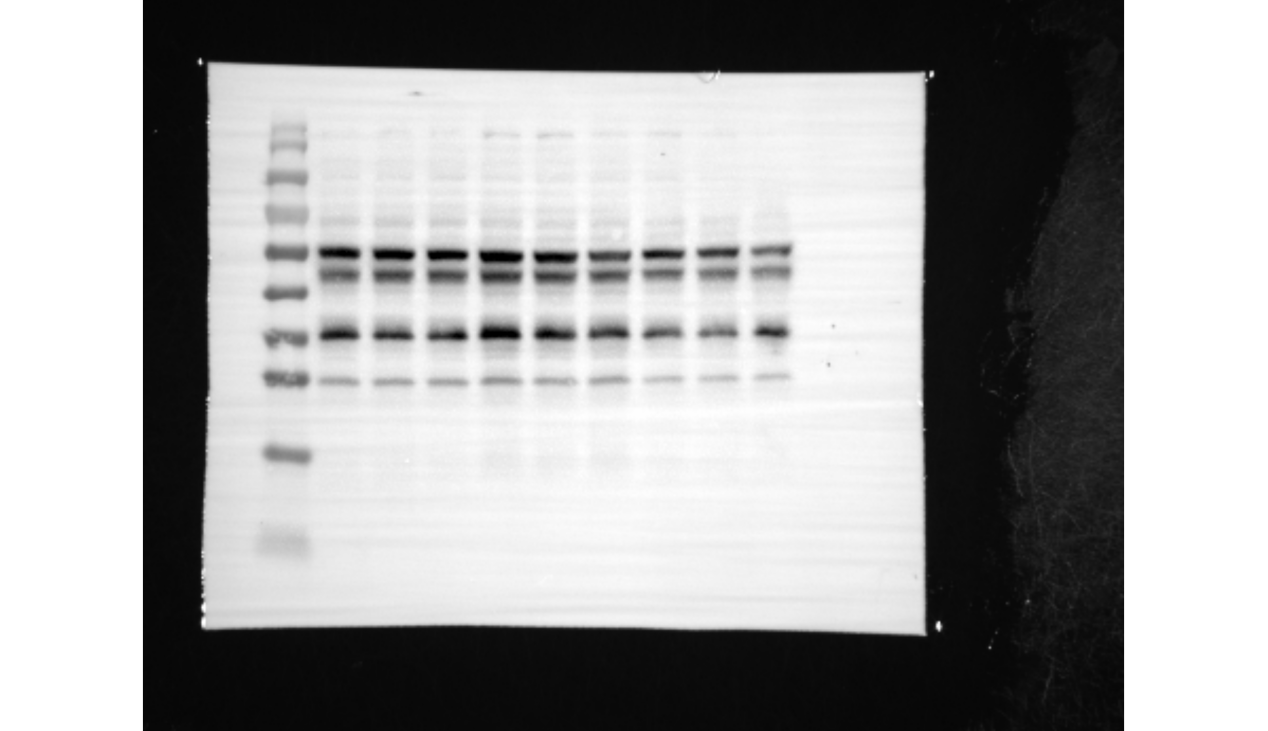


Caspase3


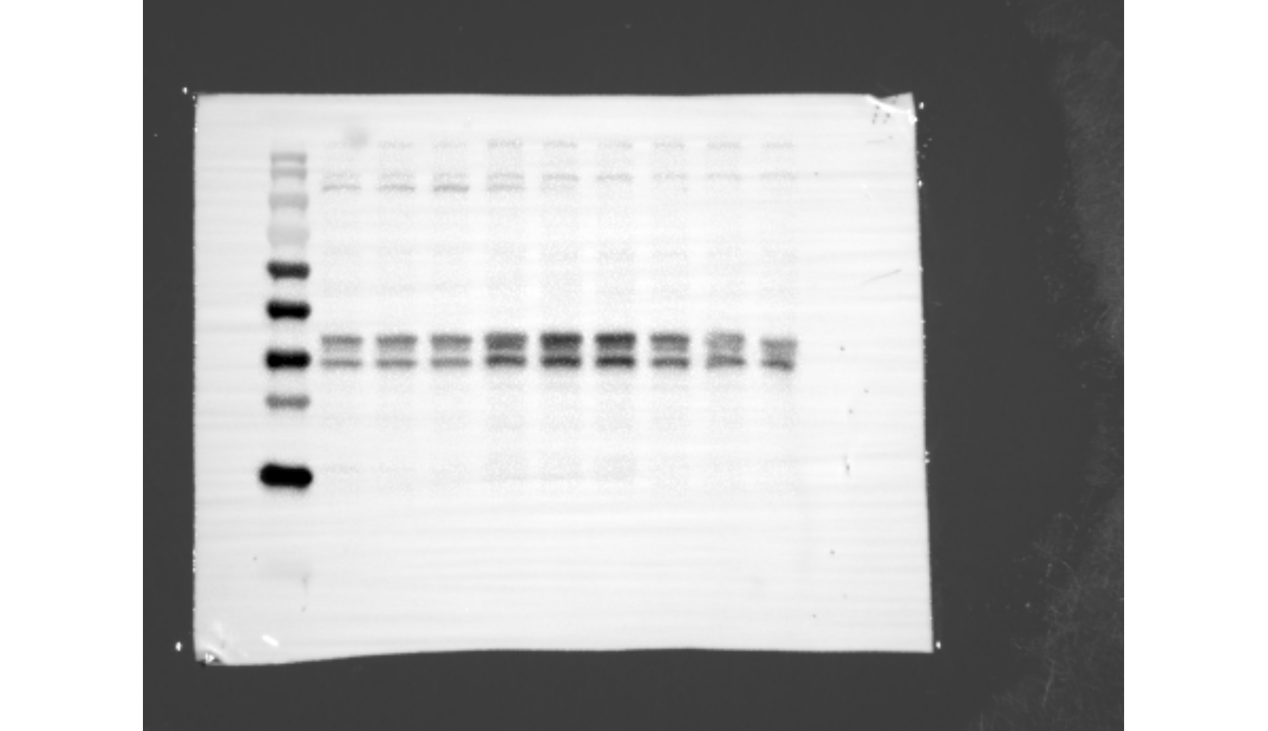

Supplement: S1 Raw images — (DOCX) [file pone.0310535.s001.docx]
