# Supplementary material for: Exploring the effects of hypoxia and reoxygenation time on hepatocyte apoptosis and inflammation
Source: PLoS One. 2024 Nov 21;19(11):e0310535. doi: 10.1371/journal.pone.0310535 (PMC11581257; doi:10.1371/journal.pone.0310535)
Supplement: Attachment — Submitted filename: Comments_1.docx [file pone.0310535.s003.docx]

I have reviewed the manuscript by Shao et al., titled "Exploring the Effects of Hypoxia and Reoxygenation Time on Hepatocyte Apoptosis and Inflammation." This study aims to develop an in vitro model for Hepatic Ischemia-Reperfusion Injury (HIRI), a critical concern during liver surgeries such as transplantation and resection. The researchers utilized the human liver cell line HL-7702 to simulate HIRI, employing a hypoxia chamber to mimic ischemic conditions followed by reoxygenation. They observed that inflammation and apoptosis levels in the cells increased with prolonged hypoxia (6, 12, 24 hours), reaching a peak at 24 hours when coupled with a fixed 12-hour reoxygenation period.

1. The authors mention that the HL-7702 cell line, a typical human liver cell line, was preserved in the lab and revived. The specific passage number of these cells should be included for clarity and reproducibility.
2. The rationale for selecting the HL-7702 cell line warrants further explanation. This cell line may not fully capture the complex interactions of various liver cell types (e.g., hepatocytes, Kupffer cells, endothelial cells) and the broader physiological context of HIRI in humans.
3. The method used to confirm cellular hypoxia after exposing the cells to a gas mixture containing 95% N2 and 5% CO2 should be detailed. How was successful hypoxia induction verified in these cells?
4. In Figure 1, the morphology of hypoxic and normoxic cells is presented at different time intervals. What observations were made regarding the control (normoxic) cells during these intervals? The images suggest the control cells were fully confluent, and the depicted morphological changes might be common regardless of oxygenation conditions. Were specific biomarkers used to validate these morphological observations?
5. In Figure 2, the expression of TNF-alpha via Western blotting appears inconsistent across different time points. Could the authors comment on this finding, especially in the context of expected variations with time?
6. The justification for using two different housekeeping proteins in Figures 2 and 3 during Western blotting should be provided. What was the rationale behind this choice?
7. Figure 4: Western blotting experiments show signs of improper protein loading. The figure might benefit from adjustments to improve readability and accuracy.
8. While the study emphasizes inflammation and apoptosis as key markers of HIRI, other crucial processes such as oxidative stress, autophagy, and mitochondrial dysfunction might also play significant roles. The authors could consider discussing these aspects to provide a more comprehensive view of HIRI mechanisms.
9. Significant improvements in English and corrections of typographical errors are required throughout the manuscript.
